# The Pharmaceutical Technology Approach on Imaging Innovations from Italian Research

**DOI:** 10.3390/pharmaceutics13081214

**Published:** 2021-08-06

**Authors:** Giorgia Ailuno, Rosa Maria Iacobazzi, Antonio Lopalco, Sara Baldassari, Ilaria Arduino, Amalia Azzariti, Sara Pastorino, Gabriele Caviglioli, Nunzio Denora

**Affiliations:** 1 Department of Pharmacy, University of Genova, Viale Cembrano 4, 16148 Genova, Italy; ailuno@difar.unige.it (G.A.); baldassari@difar.unige.it (S.B.); 2 Laboratory of Experimental Pharmacology, IRCCS Istituto Tumori “Giovanni Paolo II”, O. Flacco St., 70124 Bari, Italy; r.m.iacobazzi@oncologico.bari.it (R.M.I.); a.azzariti@oncologico.bari.it (A.A.); 3 Department of Pharmacy-Pharmaceutical Sciences, University of Bari “Aldo Moro”, Orabona St. 4, 70125 Bari, Italy; antonio.lopalco@uniba.it (A.L.); ilaria.arduino@uniba.it (I.A.); 4 Nuclear Medicine Unit, S. Andrea Hospital, via Vittorio Veneto 197, 19124 La Spezia, Italy; sara.pastorino@asl5.liguria.it

**Keywords:** imaging, diagnosis, nanosystem, nuclear medicine, radiotracers, formulations, tumor diagnosis, tumor imaging

## Abstract

Many modern therapeutic approaches are based on precise diagnostic evidence, where imaging procedures play an essential role. To date, in the diagnostic field, a plethora of agents have been investigated to increase the selectivity and sensitivity of diagnosis. However, the most common drawbacks of conventional imaging agents reside in their non-specificity, short imaging time, instability, and toxicity. Moreover, routinely used diagnostic agents have low molecular weights and consequently a rapid clearance and renal excretion, and this represents a limitation if long-lasting imaging analyses are to be conducted. Thus, the development of new agents for in vivo diagnostics requires not only a deep knowledge of the physical principles of the imaging techniques and of the physiopathological aspects of the disease but also of the relative pharmaceutical and biopharmaceutical requirements. In this scenario, skills in pharmaceutical technology have become highly indispensable in order to respond to these needs. This review specifically aims to collect examples of newly developed diagnostic agents connoting the importance of an appropriate formulation study for the realization of effective products. Within the context of pharmaceutical technology research in Italy, several groups have developed and patented promising agents for fluorescence and radioactive imaging, the most relevant of which are described hereafter.

## 1. Introduction

Medical imaging is widely used in patient care and biomedical research to diagnose, monitor, or treat medical conditions. Most clinical imaging systems are based on the interaction of electromagnetic radiation with human body and the consequent generation of signals recorded by a detector. The mathematical elaboration of these signals leads to the final image used for diagnostic or therapeutic purpose [1]. Images can be obtained by either transferring energy through a subject (as in radiography and ultrasounds), detecting emissions originating within a subject (as in optical imaging and in nuclear medicine), or using an external energy source to induce signal production within the subject (as in magnetic resonance imaging and fluorescence).

In the past, medical imaging was usually subdivided into anatomic and functional techniques, depending on their acquisition modalities and type of information produced. Anatomic imaging includes diagnostic techniques able to locate and delineate structural changes, whereas functional imaging involves imaging modalities that provide information on the functionality and metabolic processes of organs and tissues. Improved knowledge of disease mechanisms through genomics, proteomics, and animal modeling, along with advances in chemistry and molecular biology, has led to the introduction of a new field of imaging, known as molecular imaging. This type of medical imaging is defined as the ability to visualize and quantitatively measure the function of biological and cellular processes in vivo, including information on the receptor number, signal transduction aberrations, and enzymatic activities. Molecular imaging methods generally exploit radiolabeled agents with specific physical and chemical properties to detect biochemical markers or enzymatic reactions. While anatomical imaging plays a major role for diagnosis, surgical guidance, treatment monitoring, and follow up, molecular imaging aims to achieve earlier diagnosis and patient-specific therapeutic treatment (personalized medicine).

### 1.1. Imaging Techniques Most Frequently Used in the Clinical Practice

*Magnetic resonance imaging (MRI)* is a non-invasive diagnostic technique based on the interaction between an externally applied electromagnetic field and a biological tissue to visualize its structural and functional information [2]. MRI is endowed with high spatial resolution and is considered one of the best diagnostic tools in medicine. It is broadly applied to the detection of different types of cancer [3], such as prostate and breast cancer [4,5]; moreover, it is often used in neuroimaging related to the study of neurodegenerative diseases [6], schizophrenia [7], autism [8], and the organization of language in the brain [9]. However, low sensitivity and insufficient contrast are drawbacks of this technique, which indeed requires the use of contrast agents. Among them, the most frequently employed are gadolinium (Gd)-based ones, but the main issue with such contrast agents is the potential release of Gd and its consequent accumulation in liver, spleen, kidneys, heart, skin, and bladder, especially in patients with impaired kidney function. Therefore, some Gd-based contrast agents were withdrawn from the market by regulatory agencies. Therefore, new MRI contrast agents are being investigated: for example, a manganese (II) complex with dipyridoxyl diphosphate was approved by the FDA under the commercial name of Teslascan^®^; two formulations based on superparamagnetic iron oxide nanoparticles (SPION), ferumoxide (Feridex^®^) and ferumoxsil (Gastromark^®^), are also approved for liver and gastrointestinal imaging, respectively [10]. Currently, these SPION formulations, although not widely used, are a notable potential platform, e.g., for the development of multimodal imaging probes.

Different imaging techniques are based on X-rays, which are a type of ionizing radiation with wavelength between 0.01 and 10 nm [11]. These techniques are based on the absorption and scattering of an X-ray beam during its passage through tissues. Conventional X-ray radiography involves a stationary source and a planar detector [12]. The X-ray beam passing through the body is differently attenuated by the various tissues; thus, the obtained image is a “negative” of the body part to be visualized, so, because of the different attenuation coefficients of muscle, bone, fat, and other tissues, X-ray radiography provides important diagnostic information. Indeed, this technique is frequently used to detect bone fractures, calcifications, dental problems, some kinds of tumor or abnormal masses, pneumonia, and foreign objects. One of the most diffused radiographic exams is mammography, which is practiced for the early detection of cancer in asymptomatic women, to reveal suspicious breast lesions and to assess the recurrence of malignancies [13].

In computed tomography (CT), a narrow X-ray beam rotates around the patient’s body, producing X-ray projections from different orientations that are processed by a computer, producing cross-sectional images of the body, which are stacked to give a final 3D image [14]. CT is frequently used to perform imaging of several tissues and organs such as the cardiovascular system, the gastrointestinal and renal tract, lungs, liver, bone, cartilage, and tumors [11]. Contrast agents might be employed to better distinguish structures of the body having similar attenuation coefficients and to improve the quality of the CT image [12]. For example, contrast agents are usually used to clearly evidence blood extravasations or to opacify the cavity of the gastrointestinal organs. Commonly used CT contrast agents can be divided in iodine-based and lanthanide-based, while gold nanoparticles and other metallic contrast agents (based on bismuth, tantalum, iron/platinum, and barium) are being investigated.

*Fluoroscopy* is an X-ray technique that allows obtaining real-time moving images of the tissue or organ observed. Iodine and barium are commonly used as fluoroscopy contrast agents; gadolinium or carbon dioxide are used in the case of iodine allergy [15]. This technique is commonly applied to perform angiography, to visualize the gastrointestinal tract by using barium sulfate, to evaluate urinary tract abnormalities, to image uterus and fallopian tubes, and to guide the localization of catheters and implants.

*Nuclear medicine* is an imaging modality involving the use of radioactive tracers (i.e., radiopharmaceuticals). Radiopharmaceuticals are radiolabeled agents including a biologically active molecule, drug, or cell, that targets a specific organ, tissue, or a subcellular target [16,17,18], and a radioactive isotope (radionuclide) that gives off ionizing radiations. Single photon emission computed tomography (SPECT) and positron emission tomography (PET) are two non-invasive and sensitive clinical imaging techniques based on the administration of radiopharmaceuticals. These methods provide three-dimensional representations of the radiopharmaceutical distribution within the body and allow visualizing the perfusion and functionality of specific tissues. Furthermore, they can be used for therapeutic purpose, aiming to treat cancer or over-functioning organs. PET and SPECT disadvantages are radiation exposure, limited spatial and temporal resolution, and high cost. In order to overcome the low spatial resolution, hybrid techniques, such as PET/CT and SPECT/CT, have been introduced [19]: these imaging modalities permit combining the metabolic and molecular information of PET and SPECT with the anatomical detail of CT.

*Optical imaging* is a non-invasive technique using light and photon properties to provide detailed images of organs and tissues, as well as smaller structures, including cells and molecules. Light is normally generated through the administration of fluorescent tracers or other contrast agents that alter the absorption, fluorescence, and scattering properties of tissues. Another approach, more commonly investigated in research than in clinical practice, utilizes genes encoding fluorescent proteins, such as green fluorescent protein or bioluminescent proteins (i.e., luciferases) to reveal sites of gene expression and detect biological processes in vivo. In comparison to organic dyes and fluorescent proteins, quantum dots (QDs) are emerging as a new class of fluorescent labels with improved brightness, resistance against photobleaching, and multicolor emission. Although interest in optical imaging has increased in recent years for its high sensitivity, low cost, easy accessibility, and safety, current clinical applications are limited to surface (e.g., skin) or ocular imaging, since they suffer from limited tissue depth penetration [20].

*Ultrasound (US) imaging*, also named sonography, exploits high-frequency waves, which are reflected by the body structures. This technique leads to real-time images; thus, it is frequently employed to detect the movement of organs or the blood flow. Ultrasounds are also used in the diagnosis of breast, thyroid, liver, kidney, prostate, ovarian, uterine, and pancreatic cancer, and to guide biopsies [20].

*Photoacoustic imaging (PAI)* is a hybrid technique of optical and ultrasound modalities that merges the benefits of optical resolution and acoustic depth of penetration. A nano-second pulsed laser, commonly used as a light source, induces a thermoelastic expansion of patient’s tissues and consequently generates acoustic waves detectable by ultrasonic transducers. Generally, PAI exploits the intrinsic optical absorption of human tissue chromophores such as hemoglobin, melanin, lipid, and water; however, various exogenous agents can also be used to achieve better contrast and deeper imaging. With its capacity to provide structural, functional, molecular, and kinetic information, PAI has demonstrated promising potential in a wide range of preclinical and clinical applications [21].

### 1.2. Limitations of the Imaging Tools

All the imaging techniques described in the previous section are affected by different limitations. For example, optical imaging has an associated high sensitivity and spatial resolution, but it suffers from limited tissue depth penetration (typically less than 1 cm of tissue); PET is a highly sensitive radionuclide-based imaging technique, but it is spatially limited in resolution; MRI and CT are comparatively insensitive methods of imaging but are convenient techniques and are characterized by high spatial resolution. Moreover, most of the molecular imaging agents currently used in clinical practice have a very short blood circulation time (<30 min) due to their small size and non-specific biodistribution. Hence, the development of high-contrast targeted imaging agents, with controllable circulation and biodistribution characteristics, is of fundamental importance to overcome these limitations.

In order to overcome the shortcomings of individual imaging modalities, multimodal imaging techniques have been developed [22]. Fusing the molecular information related to the disease with anatomical or functional information provides accurate knowledge of pathophysiology, combined with higher sensitivity and specificity [23]. PET/MRI, PET/CT, SPECT/CT, and MRI/optical fluorescence are examples of this type of multimodal imaging.

This article reviews examples of newly developed diagnostic agents, highlighting the importance of an appropriate formulation study for the realization of effective products. Among these are examples of engineered nanosystems with the potential to be translated into clinical applications in the near future. The cited works have been selected to showcase a range of recent advances from Italian research groups across the different areas of diagnostic medicine, with specific focus on the most recent and/or advanced works.

## 2. Main Technological Strategies and Examples of Imaging Agents Developed for Improving Diagnostic Efficiency

To improve diagnostic efficiency, several strategies, such as chemical modification, the development of prodrugs/bio-precursors, and the development of conjugates with molecules/antibodies/proteins/peptides/nanoparticles, have been explored. In the next paragraphs, these procedures will be discussed, underlining the importance of appropriate setting of formulation conditions and excipients used. Among these approaches, we focused on several examples of imaging agents developed by Italian research groups (Table 1).

The study of novel imaging strategies and the development of new imaging probes have grown in the past years worldwide, and in Italy several pharmaceutical technology researchers are active in this field.

### 2.1. Chemical Modification, Prodrugs/Bio-Precursors

Radiotracers are faced with challenges to preserve their stability in vivo, cross biological barriers, and reach the target organ, tissue, cell, or subcellular organelles in order to avoid false-positive in terms of imaging and give a correct diagnosis of a disease. In this section, we will highlight the importance of the molecular structure of a radiotracer, focusing our attention on the chemical modification of fluorine radiotracers in order to increase their metabolic stability and on labeled L-DOPA bio-precursors able to cross the blood–brain barrier (BBB) and accumulate in the brain for understanding the role and metabolism of dopamine (DA) in Parkinson’s disease (PD).

#### 2.1.1. Chemical Modification of ^18^F-radiotracers to Increase their Metabolic Stability

Radiodefluorination is one of the main metabolic degradation processes affecting ^18^F-radiotracers. The release of [^18^F]fluoride in vivo and its accumulation in bones leads to false-positives in skeleton imaging and bad signal-to-background ratio [49].

Several methods have been proposed and described in the scientific literature to avoid or decrease defluorination, and the insertion of deuterium to stabilize the C-F bond seems to be the most successful approach [49,50]. A building block approach was used, developing deuterated compounds with ^18^F-label, so that in vivo radiodefluorination reaction might decrease significantly. It has been observed that the introduction of deuterium in different positions relative to ^18^F (or ^11^C) reduces the cleavage of other parts of the molecule [51].

**Figure 1:**
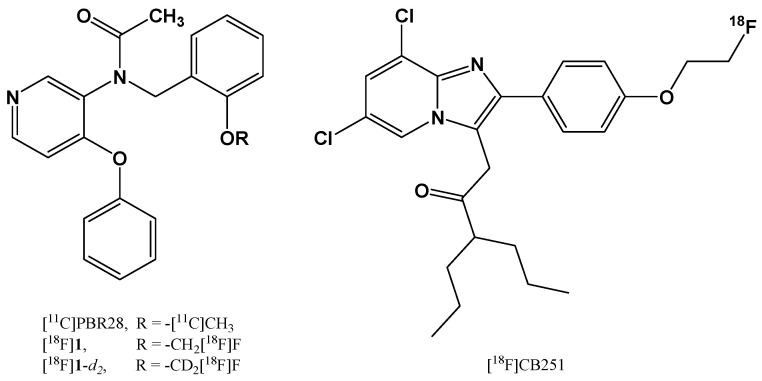
Chemical structures of [^11^C]PBR28, [^18^F]fluoromethyl-PBR28 [^18^F]1, its deuterate aryloxyanilide analog [^18^F]1-d_2_, and [^18^F]CB251.

Recently, Denora et al. from the University of Bari, in collaboration with researchers from the Department of Nuclear Medicine of the Republic of South Korea, designed and synthesized deuterium-substituted [^18^F]fluoromethyl-PBR28 (Figure 1) as a novel translocator protein 18 kDa (TSPO)-targeted radioligand with enhanced in vivo stability [24,25]. The comparison studies between [^18^F]fluoromethyl-PBR28 and its deuterated aryloxyanilide analog showed similar lipophilicity and in vitro affinity for TSPO; however, the deuterated compound provided significantly lower femur uptake in an ex vivo biodistribution study. The deuterated aryloxyanilide analog also selectively accumulated in the inflammatory lesion in an LPS-induced acute neuroinflammation rat model, comparably to the non-deuterated compound. Moreover, the compound was also tested in vivo on a mouse myocarditis model, evidencing a heart-to-lung uptake ratio 1.32-fold higher than in the healthy myocardium. The results indicated that the deuterated analog had higher in vivo stability, resulting in improved target-to-background ratio compared to the one induced by [^18^F]fluoromethyl-PBR28.

In another study Perrone et al. synthetized, by nucleophilic aliphatic substitution in a single-step radiolabeling procedure, a new fluorine-substituted ligand, named [^18^F]CB251 (Figure 1), with high affinity and selectivity for the TSPO, which is suitable for the diagnosis of neuroinflammation, neurodegeneration, and tumor progression [17]. Tissue distribution studies in healthy mice confirmed that the radiotracer was highly uptaken by TSPO-enriched tissues and reached a desirable level in the brain. Moreover, they observed a low uptake of radioactivity in the femur that indicated negligible defluorination of the radioligand and reflected the high in vitro stability recorded for [^18^F]CB251 in human serum. These results highlighted the potential use of [^18^F]CB251 as a biomarker sufficiently stable for further in vivo PET imaging studies.

In several studies, researchers demonstrated that the presence of C[^18^F]F_3_ groups in the molecule led to reduced degradation, since the strength of the C-F bond increased in the CF_3_ group. Moreover, the insertion of a [^18^F]fluorocyclobutyl moiety or other cyclic groups was favored over open fluoroalkyl chains, due to the increased steric demand and, therefore, reduced metabolism, avoiding cleavage on this position [49,52].

Binding [^18^F] fluorine to heteroatoms such as boron or silicon has been suggested as a potential strategy to obtain radiotracers with reduced radiodefluorination [53,54]. Organoboron molecules, consisting of a boron difluoride group joined to a dipyrromethene group, comprise the important class of [^18^F]bodipy dyes and offer the opportunity to use the same molecule for PET/fluorescence dual modality imaging, as well as for hybrid optical/PET imaging [55,56].

#### 2.1.2. Labeled L-DOPA Bio-Precursors as Imaging Agents

**Figure 2:**
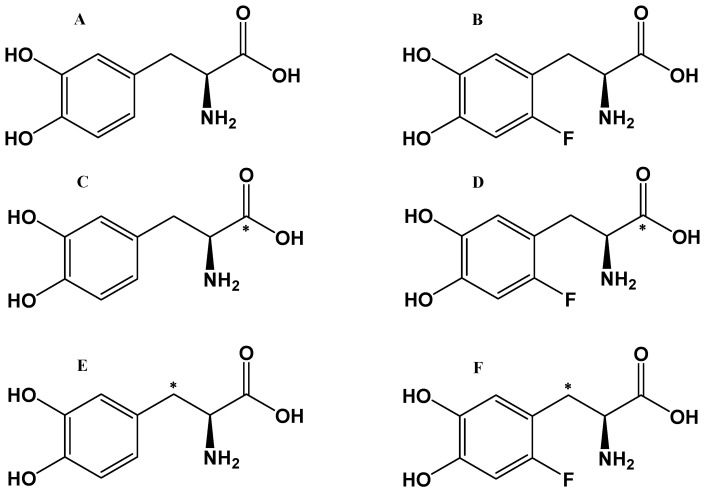
[^18^F]F-DOPA and [^11^C]-L-DOPA bio-precursor tracers. (**A**) L-DOPA, (**B**) [^18^F]F-DOPA, (**C**) [^11^C]-L-DOPA, (**D**) [^11^C]F-DOPA, (**E**) [β-^11^C]-L-DOPA, and (**F**) [β-^11^C]F-DOPA. * indicates labeling in β-position or in the carboxylic position.

Dopamine (DA) is a major central nervous system (CNS) neurotransmitter involved in numerous critical functions, including movement and cognition. Several serious diseases result from dysfunctions in the CNS DA system, such as neurodegeneration of DA neurons in Parkinson’s disease (PD). Currently, the most effective treatment for PD is represented by DA replacement therapy, and, since exogenous DA does not cross the BBB, many efforts have been spent in developing DA conjugates and prodrugs and in formulating DA-loaded nano and micro drug delivery systems that could cross the BBB and potentially treat this neurological disease [57,58,59,60,61,62,63]. The use of the amino acid L-DOPA (levodopa), the bio-precursor of DA, transported across the BBB by an amino acid transporter, is the treatment of choice in PD. Clinical trials involving L-DOPA in PD have significantly influenced the development of new PET tracers for understanding the role and metabolism of DA in this disorder. L-DOPA chemical analogues as tracers (Figure 2), and their injectable stable formulations have been developed and evaluated to assess DA turnover [26,64].

Similarly to L-DOPA, [^18^F]F-DOPA can cross the BBB by L-amino acid transporter, and its accumulation in the brain can reflect the functional integrity of the presynaptic dopaminergic synthesis [65] and enable visualization of the activity of aromatic amino acid decarboxylase (AADC), which converts [^18^F]F-DOPA to ^18^F-DA. Moreover, the [^18^F]F-DOPA uptake can be relevant for determining the effects of the treatment of the underlying pathophysiology [66].

Yet, one important disadvantage of [^18^F]F-DOPA arises from the fact that the radiotracer undergoes all the metabolic pathways of DA bio-precursor L-DOPA, including the formation of 3-O-methyl-DOPA in the periphery by the action of the catechol-O-methyl-transferase. Extracerebral 3-O-methyl-F-DOPA can cross the BBB, distribute in the brain, occupy a large volume of distribution, and consequently raise background ‘‘noise’’ in the [^18^F]F-DOPA image, diminishing image contrast and making the analysis difficult.

[^11^C] is an important PET radioisotope for pharmacological studies and has been used to label several drugs, including L-DOPA. However, its 20-min half-life represents an issue and makes the preparation of ^11^C-radiotracers more demanding [67]. In order to eliminate the complicating biochemical effects of a fluorine atom in [^18^F]F-DOPA [68], [^11^C]-L-DOPA was synthesized with the [^11^C]-label in the carboxylic position [69]. As a result of this placement, [^11^C] is released as CO_2_ upon decarboxylation by AADC [70], but the image analysis to obtain measures of AADC activity is complicated. In 1990, the synthesis of the first useful [β11-C]-L-DOPA, with the [^11^C]-label in the β-carbon position, was described [71]. In studies comparing [[β^11^-C]-L-DOPA and [β^11^-C]-F-DOPA as tracers for DA synthesis in anesthetized animals, Torstenson et al. [72] found that [β^11^-C]F-DOPA underwent more extensive 3-O-methylation compared to [β^11^-C]-L-DOPA and suggested that the high background contribution of the O-methylated product leads to underestimation of DA synthesis rates by the [^18^F]F-DOPA method when measures based on a reference tissue model are used.

### 2.2. Formulation Strategies: The Use of Buffers

Solution pH is an important factor in drug formulation, processing, drug stability, and radiolabeling. The regulation of pH is typically managed by using a buffer system, which must have a suitable pK_a_. In the following section, we will discuss some examples of the use of buffers and their importance in radionuclide complexation and the development of stable injectable [^18^F]F-DOPA formulations.

#### 2.2.1. Buffers for Gallium-68 Radionuclide Complexation

The radiosynthesis of gallium-68 (^68^Ga) molecules often requires a labelling mixture with a pH between 3.0 and 5.0 to guarantee high and fast complexation of ^68^Ga with the precursor and avoid the formation of colloids [73]. Buffers such as 4-(2-hydroxyethyl)-piperazine-1-ethanesulfonic acid (HEPES) or some carboxylic acids have been used in the labeling of radiopharmaceuticals with ^68^Ga and also other radionuclides [73]. These buffers have been reported to have low metallic complexation properties, making them appropriate to adjust pH during labeling and decrease the formation of Ga hydroxide colloids [74]. Velikyan et al. have demonstrated the beneficial effect of HEPES on the specific activity in radiolabeling bioconjugates [57,75], and several scientific works have indicated that adjusting the pH with HEPES during radiolabeling with ^68^Ga yields excellent molecular activity, reproducibility, reliability, and versatility [57,74,76,77].

**Figure 3:**
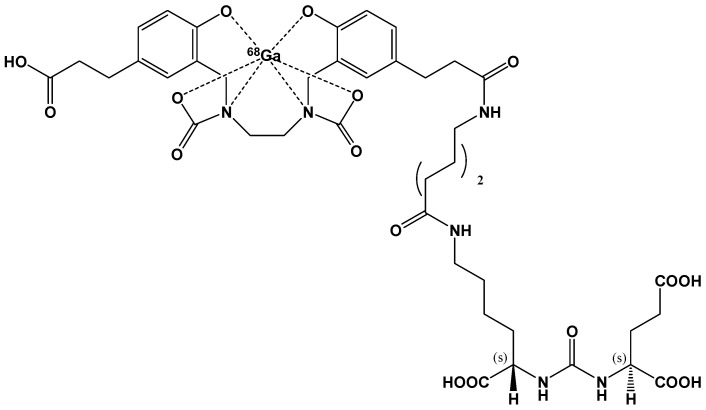
Chemical structure of the radiotracer for PET imaging [^68^Ga]GaPSMA-11.

Recently, the FDA approved [^68^Ga]GaPSMA-11 drug for PET imaging of prostate-specific membrane antigen (PSMA) positive lesions in men with prostate cancer (Figure 3).

Several cold kits for [^68^Ga]GaPSMA-11 production have been introduced in clinical studies, and recently some of them have been approved for human use in diagnosis, including those developed by the pharmaceutical company ANMI, which proved to be safe for clinical imaging without significant differences compared to [^68^Ga]GaPSMA radiopharmaceuticals produced via synthesis modules [78,79].

In order to maintain the pH of the labeling solution in a range between 3.0 and 5.0, to guarantee both a fast chelating reaction and radionuclide solubility, two cold kits have been produced using sodium acetate/acetic acid or sodium formate/formic acid buffering medium [80,81].

In a recent work, Urbanová et al. designed a new automated labeling method of PSMA-11 that minimizes chemicals use and purification steps, providing quantitative labeling yields at room temperature. They tested and demonstrated that labeling the PSMA-11 ligand with ^68^Ga in 0.1 M NaHCO_3_ (in a pH range between 7.2 and 9.0) using an SPE reversed-phase mixed-mode cation exchanger cartridge was rapid, simple, and reproducible, avoiding heating and more complex purification steps [82].

Recently, Lopalco et al. have investigated the ability of some excipients to prevent the oxidative degradation of PSMA-11 in a lyophilized formulation and guarantee fast ^68^Ga-labeling of the ligand, obtaining quantitative yields of [^68^Ga]GaPSMA-11 in a buffered solution at pH around 6.0 and laboratory temperature [83,84,85,86,87,88,89,90]. Their findings are reported in a pending patent.

#### 2.2.2. Buffers for DA Precursor Formulations

In addition to PET diagnosis of Parkinson’s disease [91], other clinical applications of [^18^F]F-DOPA have arisen in other areas of medicine, including oncology [92].

The commercial preparation of [^18^F]F-DOPA (IASOdopa^®^) employs an acidic aqueous solution (pH values around 3.0) in the presence of acetic acid to prevent the oxidative degradation of the radiotracer [93]; however, immediately before injection, the pH must be adjusted to a higher value (between 4.0 and 5.0) by adding sodium bicarbonate [94]. As further drawback, a light and transient burning sensation at the injection site has been reported as a side effect [93,95].

To possibly overcome the disadvantages related to IASOdopa^®^ injectable solution,^,^ Denora et al. developed an innovative formulation of [^18^F]F-DOPA in a sterile solution of lactate at pH 5.0, which guaranteed a significant improvement of the drug chemical stability. This pH value is compatible with the direct infusion and conferred longer shelf lives than the one reported for the commercial product [26,64].

### 2.3. Conjugates for Active Targeting

A widely investigated strategy to improve the outcomes of imaging procedures is active targeting. Indeed, by selecting a specific molecular target overexpressed by the tissue to be imaged and by conjugating the imaging probe to a molecule (i.e., an antibody, protein, or peptide) with high binding specificity for the selected target, a significant improvement in the signal-to-background ratio might be obtained. Moreover, by selectively directing the imaging agent to the target tissue, accumulation of probes in other tissues is avoided, thus reducing potential damage.

A peculiar active targeting approach is represented by pre-targeting, consisting in the decoupling of the targeting agent (usually an antibody) and a small radionuclide. Indeed, an in vivo pre-targeting procedure involves first the injection of the targeting agent, which might be followed by a chasing step to eliminate the unbound fraction, thus reducing the background signal; then, the radioligand is administered. Due to its small dimensions, the radioligand usually exhibits favorable pharmacokinetics, with rapid distribution and excretion [96].

A number of antibodies have been approved for clinical use as therapeutic agents, and the repurposing of these antibodies for imaging applications is advantageous because their biodistribution, pharmacokinetics, and potential side effects are already known [97]. Antibodies, being high molecular weight proteins, are characterized by very high binding affinity and specificity for their target, but they also exhibit long plasma half-life, which determines high and persisting blood background [98]. The long circulating half-life might determine higher accumulation of the antibody-based imaging agent at the target site, but also in the liver. To overcome the drawbacks of the excessively long half-life and to reduce non-specific immune recognition, monoclonal antibody fragments and nanobodies (i.e., single-domain antibodies) have been developed. Antibody-based imaging agents have been investigated for their potential application in the diagnosis of autoimmune diseases [99,100], cancer [101,102], and cardiovascular diseases [103].

As an alternative to antibodies, proteins and peptides can be used as targeting agents for imaging purposes, featuring some advantages over antibodies, such as lower molecular weight (allowing better tissue penetration). Moreover, peptides can be identified by high-throughput screening, are easy to manipulate due to the numerous chemical groups, and their synthesis and scale-up is cheaper [104].

Another strategy to achieve active targeting is represented by the use of polysaccharides, such as hyaluronic acid, and small molecules, such as folate [105].

In addition to the possibility to radiolabel the above-mentioned targeting molecules and to use them as such for imaging purposes, they have also been employed to decorate NPs detectable through different imaging techniques, thus combining the advantages of a nanoparticulate system with the efficacy of active targeting [106,107].

#### 2.3.1. Polysaccharide- and Albumin-Based Conjugates for Nuclear Imaging

In the context of polysaccharide-based imaging agents, Boschi et al. [27] developed an interesting class of dextran-mannose multifunctional ligands, which were conceived for binding to the [^99m^TcN]^2+^ group [108], being the Tc(V) nitrido complexes widely investigated in the search of new ^99m^Tc-radiopharmaceuticals [109]. These multifunctional ligands were conceived for sentinel lymph node detection: actually, sentinel lymph nodes are the first to receive lymphatic drainage and metastatic cells from primary tumors; therefore, their accurate identification might be crucial for establishing the tumor staging and the extension of surgical intervention. The authors synthesized and characterized a series of dextran derivatives, following different chemical procedures. The in vitro stability of the ^99m^Tc-labeled compounds was evaluated by monitoring the radiochemical purity (RCP) at different time points, and the authors observed no stability differences among the prepared multifunctional dextran derivatives. However, the authors stated that further in vivo evaluations were needed.

**Figure 4:**
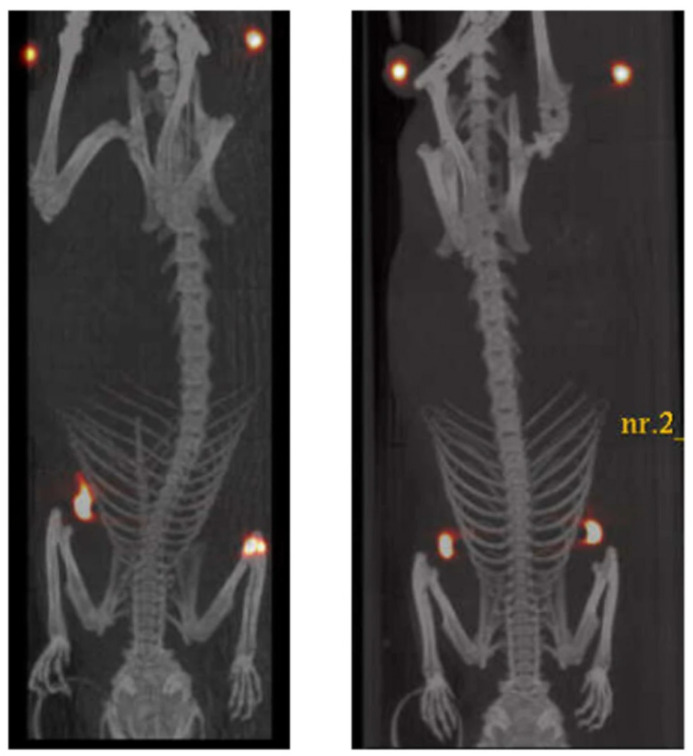
µPet/CT superimposed images of two adult female rats, 40 h after the injection of ^64^Cu-HAC in the mammary glands. In the upper part of the images, near hind legs, two objects with high density and imbibed with ^64^Cu are located as reference points for accurate overlapping of PET and CT images. Reproduced from [28] under a Creative Commons Attribution 4.0 International License (http://creativecommons.org/licenses/by/4.0/).

Among the proteins exploited for targeting purposes, albumin has recently emerged as a promising carrier for diagnostic agents [110]: Nanocoll^®^ and Albures^®^ are two formulations of Technetium-99m (^99m^Tc)-aggregated albumin approved for the diagnosis of cancer and arthritis, while other albumin-based imaging tools are still under clinical and preclinical investigation [28]. The pharmaceutical technology group at the University of Genoa developed a novel microdispersed albumin derivative (HAC) carrying a DOTA ring for radio-guided surgery of occult breast cancer lesions [28]. This new nuclear imaging agent is conceived to overcome the issues of radio-guided occult lesion localization (ROLL) employing ^99m^Tc, which is characterized by a short half-life, so that surgery should take place within 24 h from the localization of the tumor. Moreover, HAC is insoluble in aqueous media between pH 3.0 and 8.5, limiting its migration to healthy tissues due to lymphatic drainage. HAC was obtained as a white gelatinous precipitate from the one-pot conjugation reaction between purified human albumin and p-SCN-Bn-DOTA, in the form of microaggregates. The conjugate was thoroughly characterized by DSC and IR analysis; MALDI-TOF spectrometry allowed to quantify the number of DOTA per albumin molecule and the relative abundance of the different n-substituted derivatives, and the mean degree of substitution was also confirmed by a colorimetric assay [111]. The radiolabeling of HAC with Lutetium-177 (^177^Lu) and Indium-111 (^111^In) yielded products with high RCP and stability to radiolysis in saline suspension for at least 6 days after labeling. For in vivo studies, HAC was labeled with the positron-emitter Copper-64 (^64^Cu); ^64^Cu-HAC was tested on adult female rats by using a µPET/CT system, evidencing that the tracer remained located in the mammary glands for 40 h without spreading in healthy tissues or being drained by the lymphatic circulation (Figure 4). These preliminary studies highlighted that this new albumin derivative, labeled with ^177^Lu or ^111^In, might improve the efficacy and safety of the ROLL procedures; moreover, the low-penetrating beta particles emitted by ^177^Lu might be exploited for destroying tumor cells surrounding the lesion. Future studies in an authorized clinical trial might confirm the feasibility and efficacy of this new imaging agent.

#### 2.3.2. Conjugates for PET Imaging

Inflammation is involved in the onset and progression of different pathologies such as, among others, atherosclerosis. Indeed, it has been observed that, since the first stages of plaque formation, the endothelial cells near the atherosclerotic lesion begin to overexpress some inflammatory adhesion proteins such as selectins, InterCellular Adhesion Molecule-1 (ICAM-1) and Vascular Cell Adhesion Molecule-1 (VCAM-1). In particular, VCAM-1 is a very promising target for early atheroma detection, because of its low constitutive expression in healthy endotheliocytes. Therefore, different works in the literature describe a variety of systems developed for targeting VCAM-1 for both diagnostic and therapeutic purposes [112,113].

The group of Caviglioli at the University of Genoa developed two novel VCAM-1 targeted radiopharmaceuticals [29], based on the VCAM-1-binding peptide having the sequence VHPKQHRGGSKGC, which was previously discovered by in vivo phage display selection [114]. The novel imaging agents, which are able to specifically and selectively bind this adhesion protein, might be exploited for the early diagnosis of a number of inflammation-related pathologies such as atherosclerosis, some neurodegenerative diseases (such as multiple sclerosis and Alzheimer’s disease), and several types of cancer. The first radiopharmaceutical synthesized by the group, named MacroP, is based on a maleimido-monoamide DOTA derivative, having a maleimide moiety exploitable for conjugation reactions with the thiol groups of peptides and proteins: indeed, this molecule was directly conjugated with the thiol group of the cysteine of the aforementioned VCAM-1 binding peptide. The second radiopharmaceutical, named NAMP, was conceived to be exploited in a flexible three-step pre-targeting system based on the avidin/biotin high-affinity complex [30]. In the three-step pre-targeting procedure, NAMP, a biotin derivative of the same VCAM-1-binding peptide mentioned above, would be injected first; the second step would involve the administration of avidin or its derivatives streptavidin or NeutrAvidin^TM^, strongly binding NAMP biotin moiety; finally, a biotinylated radiotracer able to bind avidin would be injected. Both MacroP and NAMP were thoroughly characterized and identified by mass spectrometry, and, after labeling with ^68^Ga, preliminary in vitro tests were performed on TNF-α activated human umbilical vein endothelial cells (HUVEC), evidencing that both radiopharmaceuticals retained the VCAM-1 binding ability, with higher radiosignal uptake from TNF-α activated cells compared with non-activated control cells. However, even if both compounds resulted in promising PET radiotracers for VCAM-1 imaging, NAMP exhibited its superiority in the labeling procedure yield and PET sensitivity.

Exploiting the promising tumor-targeting ability of curcumin and its derivatives, Orteca and her group synthesized a new ^68^Ga-labeled curcumin derivative for PET imaging of colorectal cancer [31]. Indeed, in previous studies [115,116], the authors synthesized and characterized ^68^Ga-labeled complexes with curcumin, diacetyl-curcumin, and bis(dehydroxy)curcumin, but these compounds underwent rapid degradation in whole human blood. Therefore, in the following study, in order to improve the radiotracer stability, the authors conjugated curcumin to a DOTA ring following a two-step synthetic procedure. The product (named DOTA-C21) was characterized by ^1^H-NMR, ^13^C-NMR, and LC/MS, exhibiting two isomeric peaks corresponding to the diketo- and keto-enol forms. Preliminary studies were conducted by complexing DOTA-C21 with cold gallium using a Ga(NO_3_)_3_ solution (1.5:1 metal-to-ligand molar ratio), and the complex formation was confirmed by LC-MS, LC-MS/MS fragmentation, and ^1^H-NMR titration of Ga-DOTA-C21 with Ga^3+^ was performed to determine the chelation mode and binding sites. Moreover, the authors observed that after Ga^3+^ addition, DOTA-C21 fluorescence emission at 410 nm, typical of curcumin in the keto-enol form, was unchanged. Radiolabeling was performed by following two different procedures: the first one involved postprocessing of the generator and required the use of only 10 nmol of precursor, giving a yield of 97 ± 2%; in the second one, which was characterized by lower yield (60–80%), the labeling was conducted by increasing the precursor amount up to 80 nmol, without eluate processing. After purification by solid phase extraction, UHPLC was employed to measure RCP (>95%) and verify ^68^Ga-DOTA-C21 stability in phosphate buffer and human serum (more than 88 ± 3% and 85 ± 3%, respectively, after 120 min), while partial degradation occurred in whole human blood (the intact compound ranged from 74 ± 2% after 10 min and 57 ± 3% after 120 min). An in vitro study on HT29 colorectal cancer cells demonstrated a time-dependent uptake of ^68^Ga-DOTA-C21, reaching a radioactive uptake of 121 ± 4 kBq after 60 min incubation, which was significantly higher than the radioactive uptake of control cells receiving ^68^GaCl_3_. The authors also performed efflux studies, evidencing that after 60 min approximately 75% of ^68^Ga-DOTA-C21 remained within the cells. In vivo PET/CT imaging of HT29 tumor-bearing mice treated with ^68^Ga-DOTA-C21 evidenced that, after both intravenous (i.v.) and intraperitoneal (i.p.) injection, the tumor was immediately visible, and after 1 h tumor accumulation was 2.27 ± 0.85% and 1.89 ± 0.45%, with a tumor-to-muscle ratio of 1.91 ± 0.47% and 3.59 ± 2.76% for i.v. and i.p. injection, respectively. Regrettably, high radioactivity in the heart was also observed 1 h after the administration (5.8 ± 0.7%). Ex vivo biodistribution studies confirmed these results.

### 2.4. Nanoparticles (NPs) as Imaging Agents

Different types of NPs are emerging as powerful tools for molecular imaging. Indeed, NPs offer several advantages as imaging probes, because of their controllable physical properties, facile surface modifications and enhanced stability, circulation time, and tumor accumulation, in comparison with small-molecule and antibody-based imaging probes [117,118]. Various materials can be employed in NPs production, thanks to their peculiar properties suitable for generating a measurable signal.

#### 2.4.1. Nanoparticles as MRI-Imaging Agents

Cavalli and her group at the University of Torino recently developed chitosan nanobubbles with a liquid perfluoropentane core for the co-delivery of the drug prednisolone phosphate and the MRI agent Gd(III)-1,4,7,10-tetraazacyclododecane-1,4,7,10-tetra(methylene phosphonic acid) (DOTP) complex, thus obtaining a theranostic nanosystem [40]. The nanobubbles were prepared by mixing an ethanolic solution of Epikuron^®^ 200 with perfluoropentane, followed by the addition of water and, after homogenization, of chitosan. Two formulations, differing for the type of surfactant (palmitic and tetradecylphosphoric acid), were developed, employing Pluronic F68 as a stabilizing agent. Gd-DOTP loading was achieved by the drop-wise addition of an aqueous solution of the complex. As a result of their better properties, the nanobubbles containing palmitic acid were selected for the prednisolone phosphate loading studies, which were obtained by dissolving the drug in the ethanolic solution of Epikuron^®^ 200, resulting in 7% *w/w* drug loading. Interestingly, the authors observed that the loading of Gd-DOTP and prednisolone caused a decrease in both size and zeta potential. The nanobubbles released prednisolone quickly under ultrasound stimulation, while without stimulation only 2% of the drug was released after 1 h. Moreover, in vitro measurements evidenced that the nanobubbles were detectable by both echography and MRI. This study represents an interesting proof of concept for the feasibility of nanobubbles as theranostic agents.

By combining multiple functions in a single NP, it is possible to develop new versatile nanosystems allowing, for example, multimodal imaging or theranostic applications. A possible way to obtain multifunctional NPs is represented by bi-metallic NPs. In this context, Torresan et al. [41] developed, by an easy, one-step, laser-assisted synthetic procedure, iron–boron (Fe–B) NPs coated with a shell of polyvinylpyrrolidone. The obtained NPs, exhibiting an average diameter of 26 ± 15 nm as measured by dynamic light scattering (DLS), featured the functions required for both neutron capture therapy (NCT) and MRI. The authors demonstrated that the Fe–B NPs exceeded by three orders of magnitude the payload of boron isotopes contained in clinical sensitizers. Moreover, through in vitro cytotoxicity tests performed on different cell lines (L929 fibroblasts, 4T1 mammary carcinoma cells, and B16 melanoma cells), the authors demonstrated that Fe–B NPs were biocompatible, undergoing slow degradation in the lysosomal environment. Finally, the Fe-B NPs in vivo biodistribution in healthy mice was monitored by MRI, evidencing longer circulation time and reduced spleen accumulation, compared to the commercially available Endorem. These novel Fe-B NPs represent a promising tool to optimize the boron–NCT procedure, by performing 3D image visualization of boron distribution prior to neutron irradiation.

In the context of nanoparticles properly designed for the use as MRI contrast agents, it is worth mentioning the work of Truffi et al. [43]. They prepared poly(lactic-co-glycolic acid)-block-b-poly(ethyleneglycol) (PLGA-PEG) polymer micelles targeted to the endothelial mucosal addressin cell adhesion molecule 1 (MAdCAM-1), conjugated with the half-chain of a monoclonal MAdCAM-1 antibody. The loading with QDs as a stable fluorescent tracer allowed using them to selectively localize the inflammatory bowel disease (IBD) foci in a preclinical experimental model of IBD and ex vivo in tissue samples from Crohn’s disease patients. The QDs-loaded micelles were prepared via the single emulsion method with controlled size and homogenous size distributions; their conjugation with the anti-MAdCAM-1 antibody was confirmed by DLS analysis, with a slight increase in the size of NPs which, upon further characterization by electron microscopy, resulted spherical, with around 150 nm size, showing several QDs situated within the inner core. Moreover, QDs were replaced by MnO nanocrystals to demonstrate their possible use as a novel imaging tool for MRI. The authors yet demonstrated in a previously published work the colloidal stability of MnO-loaded PLGA-PEG NPs (MnO@PLGA) and measured their relaxivity potential, showing a dose and time-dependent relaxation rate r^1^ enhancement caused by the release of Mn^2+^ ions from PLGA matrix [119]. In this study, MnO@PLGA were tested in vivo by performing MRI scans of the rectum (the lower part of the large bowel) after 24 h by the injection, revealing localized contrast-enhanced regions with correct maintenance of the anatomic background. This represents a remarkable advantage for a device to be used as contrast agent, as a result of the specific localization of functionalized NPs on MAdCAM-1-positive vessels.

#### 2.4.2. Nanoparticles as PET Imaging Agents

Piras et al. at the University of Pisa developed carboxymethylcellulose (CMC) NPs labeled with ^68^Ga for white blood cell imaging through PET [44]. The CMC-based NPs were prepared by ionotropic gelation by pursuing two different approaches. In the first kit type approach (kit-type I), ^68^Ga eluting from the radionuclide generator was used as a co-crosslinking ionotropic gelation agent for in situ formation of the NPs (*In-situNP*), while in the second approach (kit-type II), the previously prepared CMC-NPs were labeled with ^68^Ga. To prepare the kit-type II NPs, 0.05 N HCl was added to a CMC solution, followed by CaCl_2_, acting as the ionotropic agent, while in the case of the *In-situNP*, the freshly eluted ^68^Ga solution was used in place of plain HCl, in the presence of CaCl_2_. For the radiolabeling of kit-type II formulations, the NPs as suspension or powder (NPSusp and NPLyo, respectively) were incubated with a ^68^GaCl_3_ 0.05 N HCl solution, evaluating the effect of different NP concentrations and incubation times. NP formation was confirmed by ATR/FT-IR analysis, and *In-situNP* exhibited a lower mean hydrodynamic diameter (206.0 ± 6.7 nm) compared to *NPSusp* and *NPLyo* (299.5 ± 4.3 nm and 342.4 ± 5.1 nm, respectively), which was measured by DLS. Interestingly, the labeling efficiency of *In-situNP* (11.4%) was consistently lower than *NPSusp* and *NPLyo* ones (81% and 83%, respectively). The authors studied the pH stability profile of NP size, observing a progressive loss of stability at lower pH values, due to the crosslinking reduction and to CMC insolubility. The serum stability was also evaluated in order to set the optimal incubation conditions with white blood cells and limit the coagula formation due to NP interaction with serum proteins. White blood cell internalization of labeled *In-situNP*, *NPSusp,* and *NPLyo* was studied by evaluating the cell radioactivity uptake and time persistence. Only *NPLyo* passed both the visual inspection and trypan blue exclusion test, suggesting a time-dependent uptake; moreover, to confirm that the radioactivity recovery from cells was due to NP internalization, the authors repeated the experiment using fluorescein isothiocyanate (FITC)-labeled *NPLyo* and observing the fluorescent uptake through confocal microscopy. On the contrary, *In-situNP* were scarcely internalized because of the presence of large amounts of free CMC, not contributing to cell labeling, while *NPSusp*, after internalization, formed macroscopic aggregates, compromising the success of the white cell labeling procedure. Interestingly, as CMC is a GRAS excipient, a faster clinical translation of this nanosystem might be feasible.

Rainone and his group developed multifunctional fluorescent ^99m^Tc-labeled silica NP (SiNP) functionalized with anti-HER2 (Human Epidermal Growth Factor Receptor 2) antibody in the form of a Trastuzumab-half chain (Hc-TZ) for HER2-positive breast cancer detection through SPECT [46]. SiNP were prepared by Stöber synthesis [120], which was followed by decoration with multiple functionalities, leading to a fluorescent core–shell structure engineered with a biocompatible PEG coating, a targeting function (Hc-TZ, whose presence was confirmed by Dot Blot analysis), and a complex of (3-isocyanatopropyl)triethoxysilane, IPTES, with nitrilotriacetic acid, NTA, as chelating groups. The NPs showed a size of 114.3 ± 0.7 nm (PDI 0.084 ± 0.001) and a zeta potential of −33.6 ± 6.0 mV. The in vitro binding efficiency and selectivity was assayed through flow cytometry and fluorescence microscopy on SK-BR-3 cells, HER2-positive (HER2^+^) breast cancer cells, and HER2-negative (HER2^-^) MDA-MB-468 cells, using SiNP-NTA-TZ and partially functionalized NPs (SiNP-NTA, SiNP-TZ, and SiNP). The authors observed a remarkable time-dependent increase of fluorescence signal after the incubation of SK-BR-3 cells with SiNP-TZ and SiNP-NTA-TZ, compared to SiNP-NTA and SiNP, with a significantly higher uptake compared to MDA-MB-468 cells. The SiNP-NTA-TZ were labeled with ^99m^Tc, obtaining an RCP of 42%, assayed through HPLC. A kinetic binding assay evidenced that SiNP-NTA-TZ exhibited a 1.6% and 2.3% SK-BR-3 uptake after 1 and 4 h incubation, significantly different from the controls (*p* < 0.05). Finally, an ex vivo biodistribution study performed on an SK-BR-3 tumor mouse model evidenced higher tumor accumulation for TZ-functionalized NP compared to SiNP-NTA. Interestingly, the authors evidenced a reduction in tumor radioactivity concentration in animals receiving SiNP-NTA-TZ, but not in those injected with SiNP-NTA; this was probably due to the active targeting followed by cellular internalization and lysosomal degradation concerning SiNP-NTA-TZ.

In a following work [46], to overcome the limitations of therapies currently used in clinics to treat HER2-positive breast cancer, the same research group explored the potential theranostic use of fluorescent SiNP-TZ directly labeled with ^99m^Tc on the histidine residues of Hc-TZ and loaded with the anticancer drug doxorubicin (Dox). The authors prepared two formulations characterized by SiNP:Hc-TZ ratios of 1:2 and 1:8, investigating the effect of the different targeting agent density on the NP surface; radiolabeling was performed by incubating [^99m^Tc(H_2_O)_3_(CO)_3_]^+^ with a suspension of SiNP-TZ, obtaining an RCP of 19.8% by HPLC analysis. An ex vivo biodistribution study on a HER2^+^ mouse model employing ^99m^Tc-SiNP-TZ with different NP:targeting agent ratios revealed that the 1:8 ratio led to higher tumor uptake, as confirmed by fluorescent microscopy. Moreover, an in vivo preliminary study was performed on SK-BR-3 tumor-bearing mice to evaluate the distribution kinetics of ^99m^Tc-SiNP-TZ (1:8) through SPECT, showing rapid radioactivity accumulation in the tumor after 1 h, remarkably increasing at 4 h. Regarding the therapeutic purpose, Dox was externally grafted on the surface of non-radiolabeled SiNP-TZ by exploiting an IPTES-Dox complex [121], obtaining a loading of 1.5–1.6%, assayed by UV-Vis spectrophotometry. The NP were characterized for their size by DLS, evidencing an increase of both the mean hydrodynamic diameter and PDI, compared to SiNP-TZ (83.4 ± 17.3 nm and 76.97 ± 4.97 nm, respectively; 0.114 ± 0.020 and 0.384 ± 0.009, respectively). SK-BR-3 cells incubated with Dox-SiNP-TZ exhibited higher drug uptake compared to the controls (HER2^-^ cells and non-targeted Dox-loaded SiNP). In vivo optical imaging observation of Dox-SiNP-TZ injected in breast cancer xenografted mice evidenced a Dox accumulation in the tumor comparable to Caelyx^®^, which is a commercially available liposomal formulation of Dox. Moreover, the authors observed higher treatment efficacy in vivo, which was measured as tumor volume regression and tumor growth inhibition, in mice receiving Dox- SiNP-TZ compared to Caelyx^®^. Finally, investigations on the proteomic profiling and treatment response suggested a potential benefit of DOX-SiNP-TZ for therapeutic application in HER2^+^ breast cancer.

#### 2.4.3. Nanoparticles as Multimodal Imaging Agents

Many research groups explored the combination of different imaging techniques as a valuable strategy to fuse molecular, anatomical, and functional information.

In the field of multimodal imaging, Piccionello et al. [47] developed photoluminescent magnetic NPs combining the high time resolution and sensitivity of optical imaging with the optimal deep tissue contrast property of MRI. By following two different procedures, the authors fluorescently labeled dopamine (DA) with 7-nitrobenzofurazan (NBD); then, DA-NBD was used to decorate the surface of Fe_3_O_4_ NPs, exploiting the strong interactions between the catechol functional groups with the oxides on the NP surface. The so-obtained Fe_3_O_4_-DA-NBD NPs, analyzed through transmission electron microscopy (TEM), showed an average diameter of 3–5 nm; these NPs were further embedded in polymeric micelles composed of PLGA-PEG in order to enhance the biodistribution and biocompatibility of the system, conferring longer half-life to the fluorescent dye in the blood circulation. The polymer-embedded PL-MNPs (Fe_3_O_4_-DA-NBD@PNPs) were obtained by the nanoprecipitation method and were characterized by DLS and TEM, exhibiting an average diameter of 122 nm and a zeta potential of −25 mV. The iron content, measured by atomic absorption spectroscopy, was equal to 15 mM. Fluorescence studies after excitation at 470 nm highlighted that, while Fe_3_O_4_-DA-NBD NPs retained a strong emission band centered at 548 nm as NBD-DA, Fe_3_O_4_-DA-NBD@PNPs exhibited only a weak fluorescent emission at 548 nm for the quenching effect exerted by PLGA-PEG on the NBD fluorophore. Moreover, the superparamagnetic properties of the NPs were studied, revealing a negative MRI contrast enhancement with a contrast efficiency of approximately 142.2. The results obtained by the authors suggest this novel fluorescent magnetic NPs as a promising multimodal imaging tool.

To combine the real-time soft tissue imaging of US with the additional functional and structural information provided by PAI, Armanetti and his group developed disassembling passion fruit-like nano-architectures (*pf*NAs) composed of silica NPs embedding dye-modified aggregates of ultrasmall Au NPs [48]. The nano-architectures were prepared by following a previously described method [122]: briefly, Au NPs were functionalized with poly(L-lysine) modified with IRDye 800CW-NHS ester; then, the so-obtained Au nanoarrays were added to a tetraethyl orthosilicate solution, leading to the formation of *pf*NAs exhibiting 118.9 ± 22.1 nm diameter and 20.2 ± 1.6 nm wall thickness; ICP-MS and spectrophotometric analyses revealed a 5% *w/w* gold/*pf*NAs and 0.42 nmol/mg IRDye/*pf*NAs content. Investigation of the in vitro PA features of the *pf*NAs showed that the minimum *pf*NA concentration detectable was 7.34 nM in water and 60 nM in blood. Moreover, to increase the significance of the in vitro US feature evaluation, the authors used metal-free NAs, which generated a high US contrast signal well-over the water background. Ex vivo PA-US imaging was performed on chicken breast samples, properly simulating the echogenicity of human tissues, which were previously injected with different amounts of *pf*NAs. This test evidenced the potential application of pfNAs in high-echogenic tissues.

Non-equilibrium Au-Fe nanoalloys, exploitable for X-ray CT combined to MRI, were developed by Torresan and her group at the Universities of Padova and Verona [22]. The aim of this work was to overcome the issue of long-term biopersistence of inorganic NPs by exploiting the 4D behavior of these nanoalloys, which are capable of changing size, shape, and structure over time, degrading into Au-rich nanocrystals smaller than 10 nm. After performing numerical simulations of the degradation process of the nanoalloys, the authors prepared the NPs following a modified procedure of laser ablation in liquid [123], shifting to the non-equilibrium region of the Au-Fe phase diagram. Different amounts of Au and Fe were employed (pure Au, Au(71)Fe(29), Au(70)Fe(30), Au(50)Fe(50)), assessed by inductively coupled plasma-assisted mass spectrometry (ICP-MS). The NPs were decorated with PEG chains by exploiting the Au-S bond between thyolated PEG and the NPs surface. The formation of nanoalloys, having size in the 9–18 nm range, was confirmed by X-ray diffraction, which was used also to study the nanoalloys behavior at physiological and acidic pH (mimicking the lysosomal environment), which evidenced that the alloys with higher Fe content underwent significant structural modifications. Moreover, the composition dynamics was investigated by monitoring the absorption of the nanoalloys at about 520 nm, due to the excitation of the localized surface plasmon resonance of Au, revealing that the dealloying process is composition-dependent, confirming the outputs of the X-ray diffraction analyses. Small-angle X-ray scattering and TEM analyses evidenced that the size decrease was proportional to the Fe content increase, with the Au(50)Fe(50) nanoalloys exhibiting the more evident size change, which is quicker at acidic pH. Interestingly, the 4D behavior is triggered by iron-complexing species, such as disodium ethylenediaminetetraacetic acid (EDTA). Through FT-IR, the authors also proved that the size and structure evolution implied the loss of the PEG shell. After selecting the Au(50)Fe(50) nanoalloys as the most promising for nanomedicine applications, in vivo blood circulation and biodistribution in healthy mice was monitored through CT imaging: compared to PEGylated Au NPs, the Au(50)Fe(50) nanoalloys exhibited longer circulation time, without rapid clogging of mononuclear phagocyte system (MPS) organs or kidneys, with the contrast decreasing in all organs, due to the progressive degradation of NPs into smaller fragments. Moreover, the contrast enhancement after 78 days in spleen and kidneys suggested the elimination of the nanoalloys through glomeruli filtration, which was possible because of the 10 nm size of the NPs. Finally, the long-term fate of the Au(50)Fe(50) NPs in healthy mice was investigated by MRI, showing a clear contrast enhancement in the liver after 1 h, which decreased after 48 h; the signal dynamics showed an opposite trend in spleen and kidneys, corroborating the hypothesis of renal excretion. Histopathological evaluation revealed no toxic effects of the nanoalloys on liver, spleen and kidneys, and bidimensional energy dispersive spectra on histopathological specimens suggested that the nanoalloys degradation mainly occurred in the spleen, while the small Au-rich nanocrystals generated were eliminated through the kidneys.

Among dual imaging modalities, the synergistic combination of MRI with SPECT and/or PET is likely to become the next generation of dual-modality scanners in medical imaging for accurate diagnoses, thanks to the sensitive and quantifiable signal of SPECT/PET and the high soft-tissue resolution of MRI. Moreover, since the use of SPIONs in the MRI technique could cause the accumulation of high iron loads related to their use in localized areas of the body, the identification of alternative biocompatible NPs having the same efficacy and lower iron content has become an important need. In this context, a valuable contribution was derived from Adamiano et al. [42] with the preparation of iron-doped hydroxyapatite (FeHA) NPs and their characterization as MRI imaging contrast agents along with a proof of concept of their use as scintigraphy imaging agents for PET and SPECT following the surface functionalization of FeHA NPs with [^99m^Tc]-medronic acid (MDP). In this work, Adamiano et al. compared the diagnostic properties of their FeHA NPs with those of Endorem^®^, which is an FDA-approved SPION formulation, already used for the diagnosis of hepatic focal lesions. Endorem^®^ consists of very small (<5 nm) round-shaped electron dense NPs, while FeHA NPs consist of small isometric crystals of about 5–10 nm aggregated in needle-like NPs having a length of 70–100 nm and width of 15–20 nm. FeHA and Endorem^®^ in HEPES buffer at pH 7.4 showed negative zeta potential of –44.1 ± 0.9 mV and –25.0 ± 0.2 mV, respectively, and the hydrodynamic diameter size distributions recorded for FeHA and Endorem^®^ were 179.1 ± 3.2 nm and 105.2 ± 2.0 nm respectively, with a high degree of poly-dispersity (PDI = 0.35) for Endorem^®^, due to the presence of relevant aggregates within the dextran matrix, and a lower PDI of 0.20 for FeHA. Moreover, the authors showed that, after intravenous injection in vivo in mice at a low iron dose (1 mg kg^−1^), the FeHA NPs displayed higher contrast enhancement and longer resistance in the liver, justifying these phenomena with the high transversal relaxation rate and the higher quantity of FeHA NPs that had been administered with respect to Endorem^®^ to reach the same iron dose, as the two materials vehiculated different amounts of iron (9.7 vs. 71.0 *w/w*%). FeHA NPs were found to enhance the negative contrast also in spleen and renal cortex tissues, while on the contrary, Endorem^®^ was detected in significant concentration only in the liver. Moreover, to demonstrate the potential use of FeHA NPs as multimodal MRI-PET/SPECT imaging agents, scintigraphy/X-ray fused imaging and ex vivo studies were conducted confirming the main uptake of FeHA NPs in the liver and secondarily in other organs in which other cells of the mononuclear phagocyte system (MPS) are located, as detected by MRI. Further in vitro and in vivo studies on FeHA long-term cytotoxicity along with in vivo validation of SPECT MRI detection are necessary; however, these NPs seem to be promising imaging agents for MRI-T2 and PET-SPECT, in order to design new theranostic agents for personalized medical applications.

#### 2.4.4. Nanoparticles as Optical, Near-Infrared (NIR), and PA-Imaging Agents

Regarding the application of NP systems to the development of new optical, NIR, and PA imaging agents, several groups of Italian researchers contributed to broaden the panel of promising nanoparticulate systems for diagnostic purposes.

The use of fluorescent probes for tumor detection offers the advantage of improved biocompatibility compared to other types of contrast agents. To date, several fluorescence imaging agents based on nanodevices surface-conjugated with fluorescent dyes are reported for fluorescence biomedical imaging, especially cancer diagnosis.

**Figure 5:**
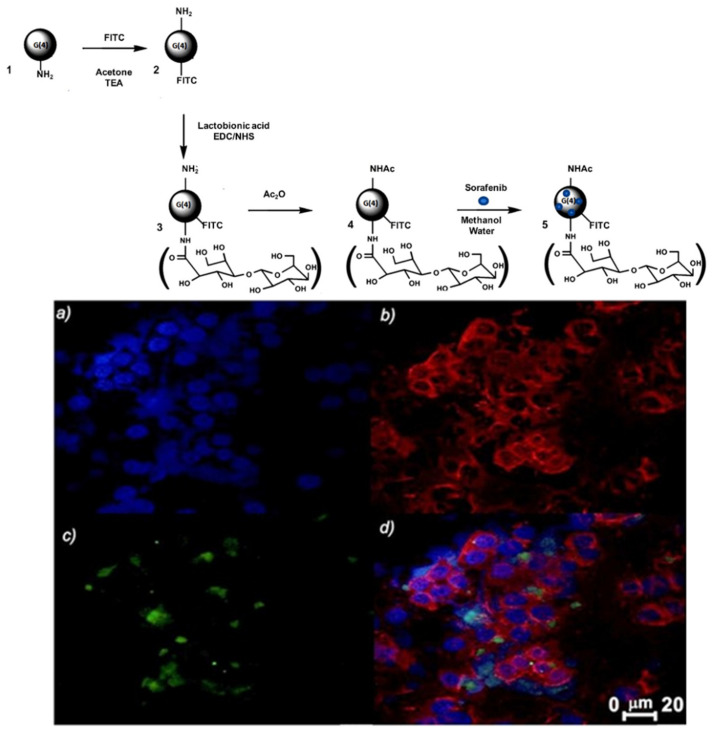
Upper panel shows the scheme of the synthesis of G(4)-PAMAM-FITC (2), Ac-La-G(4)-PAMAM-FITC (4), and Ac-La-G(4)-PAMAM-FITC/sorafenib (5) dendrimers; bottom panel shows representative single confocal slices of fixed HepG-2 cells: HepG-2 cells imaged with (**a**) DAPI; (**b**) Phalloidin-TRITC; (**c**) Ac-La-G(4)-PAMAM-FITC 4; (**d**) overlay after an incubation period of 15 min. Bar: 25 μm. Adapted with permission [32]. Copyright 2017, Elsevier B.V.

The work of the pharmaceutical technology research group of the University of Bari fits into this context: Iacobazzi et al. [32] reported the development of a fourth generation (G(4)) PAMAM dendrimer encapsulating the anticancer drug sorafenib, which is able to target, thanks to the surface lactobionic acid tag, the asialoglycoprotein receptor (ASGP-R), which is usually overexpressed in human liver cancer cell lines such as HepG2 cells. The conjugation of FITC with the targeted ASGP-R-G(4)-PAMAM dendrimer NP allowed to track and image the selective intracellular uptake in hepatic carcinoma cells. Moreover, the G(4)-PAMAM dendrimer was acetylated with acetic anhydride in order to reduce non-specific interactions with cell membranes, thus encouraging a selective uptake only by ASGP-R expressing cells. In vitro uptake studies of the cationic Ac-La-G(4)-PAMAM-FITC dendrimer (Ac = acetylated; La = Lactobionic acid) conducted by flow cytometry analysis and confocal laser scanning microscopic observations in the well differentiated human liver cancer cell line HepG-2, expressing the ASGP-R, and for comparison in HLE human liver cancer cells, characterized by undetectable level of receptor, confirmed the targeting specificity of Ac-La-G(4)-PAMAM-FITC dendrimer for ASGP-R expressing cells (Figure 5).

The same research group, in order to exploit the brain delivery of anticancer drugs and imaging of the glioblastoma multiforme (GBM), explored the use of PEG-stabilized solid lipid nanoparticles (SLNs) containing Pt(IV) prodrugs derived from kiteplatin, and they purposely designed therapeutic compounds potentially useful for the treatment of GBM [33]. The incorporation of the synthesized Pt(IV)-prodrugs in PEG-stabilized SLNs was explored to obtain nanovectors with enhanced ability to cross the BBB, which represents one of the main limitations in the therapy of brain tumors, and to accumulate the therapeutic cargo into the brain. Thus, to assess the ability of drug-loaded SLNs to permeate the BBB, optical traceable SLNs were prepared by means of co-encapsulation, in the hydrophobic core, of Pt(IV)-prodrugs and luminescent carbon dots (C-Dots) emitting in the UV–Vis region. In vitro studies, conducted on the BBB model based on hCMEC/D3 cells monolayer, proved their ability to permeate the BBB. Finally, in vitro studies conducted on U87 human glioblastoma cell line demonstrated improved cellular uptake of the Pt(IV)-prodrugs when delivered in the SLNs.

Reflecting the need for advancements in personalized medicine, some researchers also developed nanocarriers targeted to mitochondria [124,125,126,127] and useful as appropriate probes to image diseases or biological processes involving these intracellular organelles and, in particular, an interesting mitochondrial biomarker, the already mentioned TSPO protein. Variations in TSPO expression levels have been related to different diseases, from tumor to endocrine and neurological disorders; thus, this receptor has become an appealing subcellular target for the early diagnosis of a disease involving its overexpression. Targeting mitochondria is a hard and challenging task due to their highly complex structure; for this reason, the researchers of the pharmaceutical technology group of the University of Bari synthesized selective TSPO ligands having groups potentially derivatizable and therefore ideal for the preparation of targeted nanocarriers able to deliver therapeutics and diagnostics to mitochondria [62,128].

**Figure 6:**
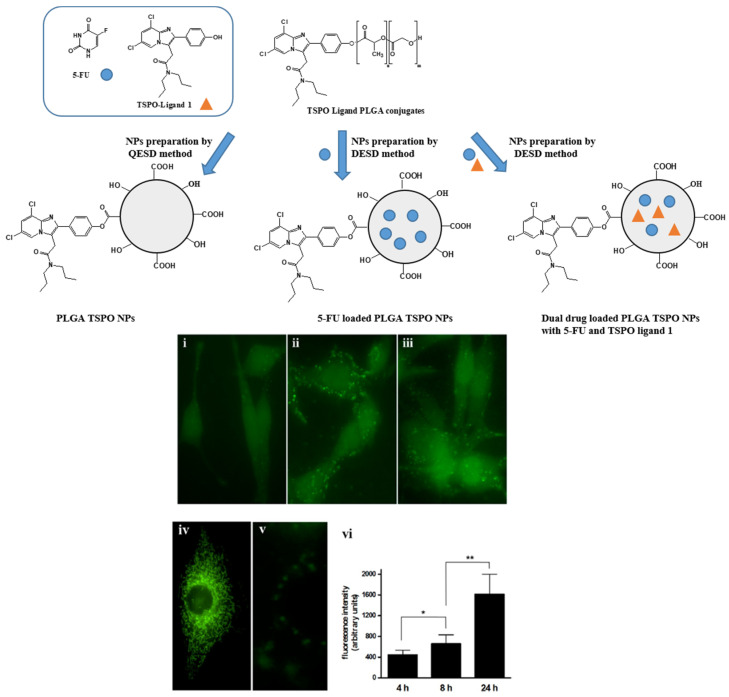
Upper panel shows a schematic representation of PLGA-TSPO NPs; bottom panel shows the fluorescence microscopy images: cells were seeded onto 24 mm coverslips and treated at 37 °C in a 5% CO_2_ atmosphere with 0.2 μM FITC−TSPO NPs (concentration is referred to FITC). Internalized FITC fluorescence was acquired after 4 (**i**), 8 (**ii**), and 24 h (**iii**). Cells incubated with 25 nM MitoTracker Red CMXRos (**iv**) and cells treatment with FITC (7 μM) alone after 24 h (**v**) were used as the control. Average intensity of internalized fluorescence of treated cells as imaged after 4, 8, and 24 h of incubation (**vi**). Data are given as means ± SD (bars) for five separate cultures. The statistical significance of differences was calculated by the unpaired Student’s t test with Bonferroni’s correction. * *p* < 0.05, ** *p* < 0.005. Adapted with permission [34]. Copyright © 2021 American Chemical Society.

Laquintana et al. [34] described the preparation of NPs employing a TSPO ligand−PLGA conjugated (PLGA−TSPO) polymer. Specifically, the PLGA–TSPO NPs (Figure 6) were prepared by a quasi-emulsion solvent diffusion procedure (QESD). In order to demonstrate the ability of TSPO targeted NPs to be internalized by cells in vitro, PLGA hydroxylic end groups were conjugated to the fluorescent probe FITC, obtaining fluorescent FITC−PLGA NPs and FITC−TSPO-PLGA−NPs. The internalization of FITC−PLGA−TSPO NPs into rat C6 glioma cells was investigated by fluorescence microscopy. As shown in Figure 6 (panels i−iii, vi), cells treated with 0.2 μM FITC−PLGA−TSPO NPs (concentration is referred to FITC) revealed a time-dependent increase of internalized green fluorescence that widely diffused in the cytosol of the cells. By contrast, no green fluorescence was imaged when cells were incubated in the presence of FITC (7 μM) (panel v), thus demonstrating that the internalized fluorescence of the cells was exclusively due to the cellular uptake of the FITC-conjugated probe. In addition, by comparing uptake studies conducted with FITC−TSPO NPs, FITC NPs, or FITC−TSPO NPs in combination with TSPO ligand 1, no significant differences were observed (data not shown), proving that, as expected, the TSPO moiety does not influence the uptake of the NPs inside cells. Since TSPO is a subcellular target, once ligands are internalized by the cells, they can bind the mitochondrial protein, inducing apoptosis.

Similarly, Lopalco et al. [35] prepared nanogels (NGs) made with TSPO ligand dextran conjugates (TSPO-Dex) and demonstrated their utility as potential delivery systems of two TSPO ligands as apoptotic agents. The internalization of TSPO ligand–dextran NGs into C6 glioma cells was studied in vitro by fluorescence microscopy. On this purpose, fluorescent TSPO ligand–dextran NGs were prepared using FITC–Dextran and TSPO–FITC–Dex NGs. Cells incubated with 5 μM TSPO–FITC–Dex NGs showed a widely diffused green fluorescence in the cytosol after 24 h. The fluorescence in cells was exclusively due to the cellular uptake of TSPO–FITC–Dex NGs because no green fluorescence was imaged when cells were incubated in the presence of 5 μM FITC–Dextran.

**Figure 7:**
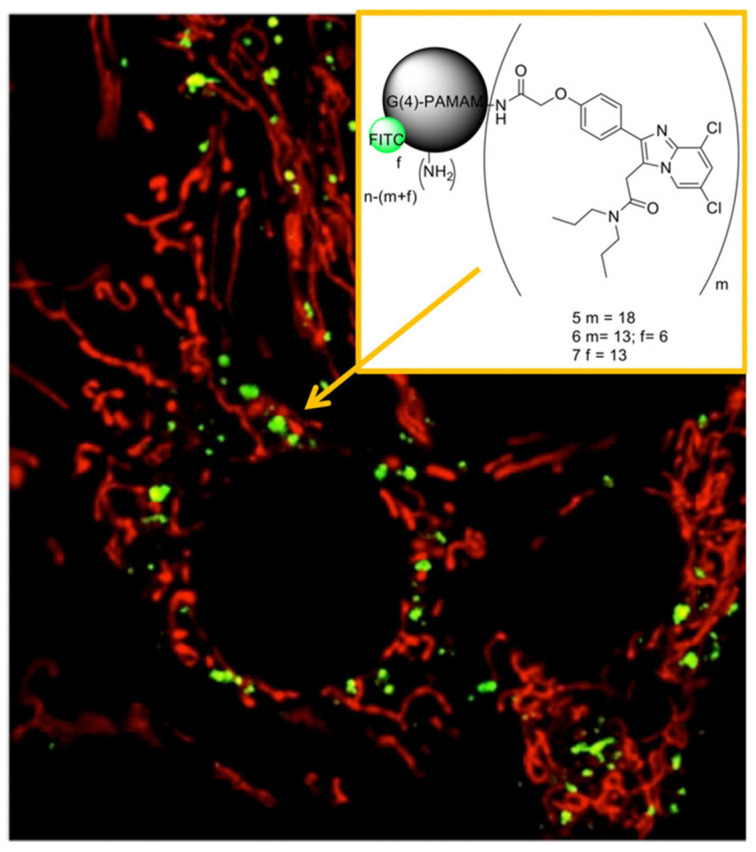
Representative confocal microscopy image of co-localization in a double-tagged C6 glioma cell (green: TSPO targeted-G(4)-PAMAM dendrimers grafted with FITC 1 μM; red: mitochondrion tagged with MitoTracker Red; yellow: convergence of red and green, indicating co-localization). Insert: TSPO ligand molecule and the TSPO-targeted G(4)-PAMAM–FITC dendrimer. Reproduced with permission [62]. Copyright 2017, Wiley-VCH Verlag GmbH & Co. KGaA, Weinheim.

Moreover, for the selective imaging of the TSPO receptor, Denora et al. [36] investigated the use of PAMAM dendrimer-based nanocarriers. The skills of synthetic dendrimers, namely the specific cellular and subcellular internalization and targeting, make them helpful to understand biological events at the molecular level. Specifically, they focused on the synthesis and in vitro evaluation of a new TSPO ligand characterized by a substituted imidazopyridine nucleus, namely the 2-(4-(6,8-dichloro-3-(2-(dipropylamino)-2-oxoethyl)imidazo[1,2-a]pyridin-2-yl)phenoxy)acetic acid (CB235), as a high-affinity conjugatable TSPO ligand linked to the amine end-group of PAMAM dendrimers, in order to obtain a new fluorescence imaging of tumor or neurodegenerative diseases overexpressing TSPO. The TSPO targeted G(4)-PAMAM dendrimers were functionalized by reacting with the organic FITC, giving fluorescent dendrimers able to bind the mitochondrial protein TSPO. The comprehensive physicochemical and morphological characterization of dendrimers by means of ^1^H-NMR, DLS, Laser Doppler Velocimetry (LDV), and Atomic Force Microscopy (AFM), as evidenced by their monodispersity, the spherical shape, and a hydrodynamic diameter of about 24 nm. To estimate the biological activity of the imaging agent, rat C6 glioma cells were incubated with the FITC dendrimers and were visualized by both confocal and fluorescence microscopy. In particular, the authors explored the cellular uptake behavior of these dendrimers in the presence of various endocytosis inhibitors, finding that the TSPO targeted-G(4)-PAMAM–FITC dendrimer was quickly internalized by C6 cells through pinocytosis and that no significant exocytosis occurred. Moreover, competition studies in the presence of the TSPO ligand, subcellular fractionation experiments and co-localization studies performed with CAT (confocal-AFM-TIRF) microscopy were crucial in confirming the ability of the TSPO-targeted dendrimer to co-localize in mitochondria (Figure 7).

**Figure 8:**
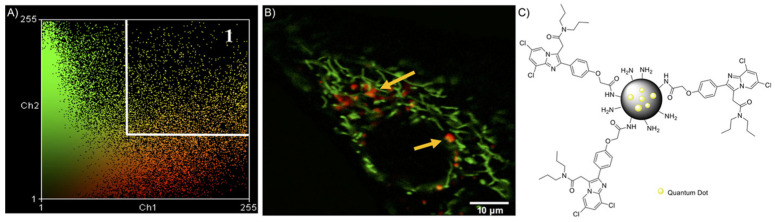
(**A**) Co-localization analysis of C6 rat glioma cells stained with MitoTracker green and TSPO-targeted QD@SiO_2_ NPs. All pixels of the co-localization image shown in panel B are reported in the scatter diagram of panel A, in which the two image channels are compared. Region 1 of the diagram displays the co-localization pixels. (**B**) Representative confocal image of co-localization of TSPO-targeted QD@SiO2 NPs (red) and MitoTracker (green) in C6 cells. The co-localization is marked by the convergence of the red and green to yellow fluorescence; scale bar: 10 μm. (**C**) TSPO-targeted QD@SiO_2_ NPs. Reproduced with permission [62]. Copyright 2017, Wiley-VCH Verlag GmbH & Co. KGaA, Weinheim.

Afterwards, the same authors in Fanizza et al. [37], in order to obviate the limitations that resulted from the use of organic fluorophores (e.g., FITC), such as broad emission spectra, susceptibility to photobleaching under continuous light exposure, and short fluorescence lifetimes, suitably engineered luminescent semiconductor QDs, based on a core–shell structure of inorganic nanocrystals (CdSe@ZnS), which are able to target mitochondria while keeping the colloidal stability and optical properties. QDs represent a new class of fluorophores that can be used as advanced photoluminescence (PL) probes, taking advantage of the QD photophysical properties and their versatile surface chemistry. Specifically, these authors prepared multifunctional nanostructures based on CdSe@ZnS QDs coated with a silica shell functionalized with amine groups and conjugated with the selective and affine TSPO ligand, CB235 (Figure 8). The silica shell allowed a significant improvement of QD stability in aqueous phase, because silica is an inert, biocompatible, and transparent material, and therefore, it is able to decrease the release of cytotoxic ions and prevent QD photo-oxidation. The nanostructure proposed in this study provides a great advantage, as it associates the photophysical stability of the QDs and the versatility of the silica shell with the TSPO receptor recognition properties of the highly affine selective ligand. The physicochemical characteristics of this new nanomaterial have been extensively studied from morphological, structural, and optical points of view; in particular, the functionalized QDs exhibited an average hydrodynamic diameter of about 50 nm and a slightly positive zeta potential of 11.7 ± 0.7 mV, determined by DLS. Finally, in vitro subcellular fractionation experiments and co-localization studies, conducted by means of laser scanning confocal microscopy on C6 rat glioma cells, showed the success of this new nanostructure to target mitochondria thanks to the molecular recognition of TSPO (Figure 8).

**Figure 9:**
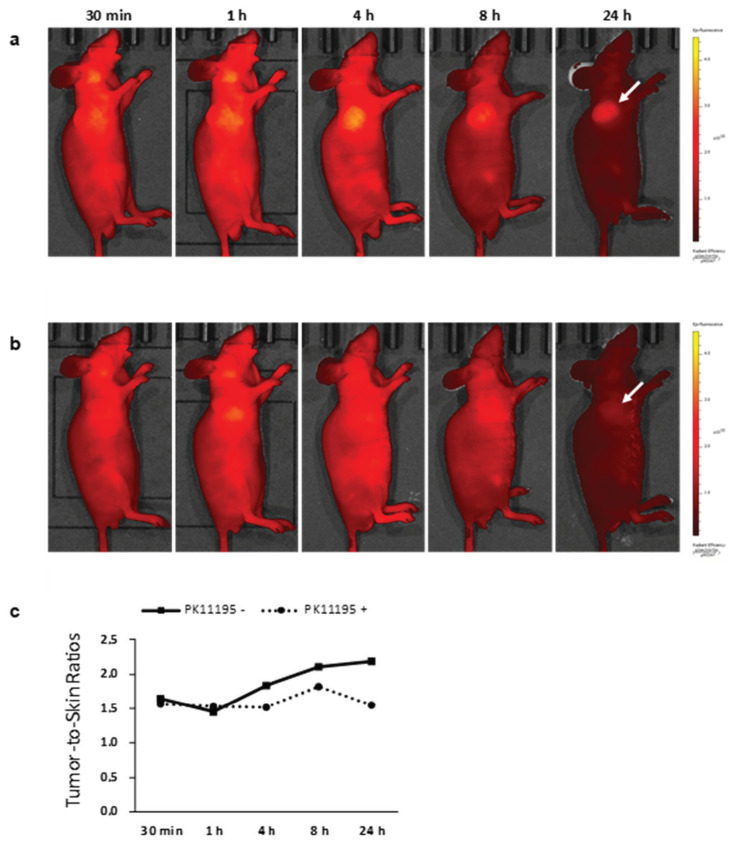
Fluorescence imaging study by IVIS Lumina XRMS. (**a**) Imaging of U87-MG xenograft model at 30 min, 1 h, 4 h, 8 h, and 24 h post-injection of TSPO-targeted NP of 200 µg. (**b**) Inhibition study using PK 11195 (10 mg/1 kg). (**c**) Tumor-to-skin ratios in mouse models with PK 11195 pre-injection or without PK11195 pre-injection. Reproduced with permission [38]. Copyright © 2021 Elsevier B.V. All rights reserved.

Denora and his group at the University of Bari also conducted an interesting research in collaboration with Professor Lee of the Department of Nuclear Medicine of Seoul National University College of Medicine (Republic of Korea), regarding the development of water-dispersible ultrasmall iron oxide nanoparticles (USPIONs) combined with an imidazopyridine-based TSPO ligand and an NIR emitting fluorescent dye [38]. The use of a fluorescent probe emitting in the near-infrared region (700−1100 nm) was justified by the enhanced signal-to-noise ratio in an in vivo technique of optical imaging. In fact, in the NIR region, autofluorescence and scattering by biological tissues are minimal; therefore, NIR probes are expected to result in high-resolution and deep penetration image. The use of targeted nanoplatform-based probes aimed to extend plasma half-lives, thus increasing the in vivo stability and targeting efficiency. The optical and structural characteristics of TSPO-targeted USPIONs were properly evaluated at each step of preparation, demonstrating the high colloidal stability in physiological media and the ability to preserve the relevant optical properties in the NIR region. The USPIONs mean hydrodynamic diameter, measured by DLS, showed only a slight increase (of about 2.6 nm) after functionalization with the TSPO ligand PK 11195, leading to a final average diameter of 5.57 ± 0.73 nm. The cellular uptake in TSPO expressing cells was assessed by confocal microscopy. The ability to recognize the intracellular receptor was confirmed in vivo by competition studies with the selective TSPO ligand PK 11195. In vivo fluorescence imaging of U87-MG xenograft models was performed to highlight the great potential of the new NIR imaging nanosystem for the diagnosis and successful delineation of GBM (Figure 9).

Personalized diagnosis and treatment with allogeneic or autologous cells are becoming a reality in the medical field. However, a crucial issue for developing the potential of this therapeutic approach and achieving a “tailored therapy” is the huge amount of cells either from autologous peripheral blood of cancer patients or immune privileged allogeneic cells. NIR-resonant gold NPs (AuNPs) hold great promise in cancer diagnosis and treatment. However, translating the theranostic potential of AuNPs into clinical applications still remains a challenge due to the difficulty to improve the efficiency and specificity of tumor delivery in vivo, as well as the clearance from liver and spleen to avoid off-target toxicity [39]. In this context fits the study of Armanetti et al. [39], in which endothelial colony-forming cells (ECFCs) have been exploited as vehicles to deliver AuNPs to tumors. The authors first demonstrated that ECFCs displayed a great capability to load AuNPs without losing viability and to exert antitumor activity per se; then, using a human melanoma xenograft mouse model, they demonstrated that AuNP-loaded ECFCs retained their capacity to migrate to tumor sites in vivo 1 day after injection and to stay in the tumor mass for more than 1 week. In addition, they demonstrated that ECFC-loaded AuNPs were efficiently cleared by the liver over time and do not elicit any sign of damage to healthy tissues. Ultimately, the study of Armanetti and his group aimed to develop an innovative vehicle of AuNPs for a theranostic approach for the diagnosis and thermoablative therapy of melanoma through the photoacoustic and tunable thermic properties of ECFC–AuNPs payload. In fact, they demonstrated in vitro that AuNP-loaded ECFCs were able to generate higher photoacoustic signals than AuNPs alone, and they also display spectral fingerprints that enabled a reliable detection of labeled cells following intravenous injection. They ascribed the enhancement of PA signal upon AuNP aggregation in intracellular vesicles to an interplay of electromagnetic and thermodynamic factors, such as heat and stress confinement.

## 3. Translational Research

A key role in the development of new diagnostic tools is played by the translational research. The translational medical research includes all the studies aiming to bridge the gap between the laboratory discoveries and the clinical application, speeding up the transfer of scientific advances into clinical practice [129], “from the bench to bedside”. Indeed, translational medicine is a precision medicine that is capable of bringing the right treatment to the right patient.

Currently, an ambitious Italian project, the ACC-GerSom project of the Fondazione Policlinico Universitario “A. Gemelli”, has been proposed as a new model of translational development [129]. The project, coordinating the work of laboratories of different scientific institutes for research, hospitalization, and healthcare, is finalized to genomic diagnostics of different types of oncology disease and, in particular, it is addressed to patients with breast, ovarian, and colon cancer [130].

Another pivotal Italian research institution, IIT—European Institute of Oncology, has launched a wide translational research program focused on the development of new diagnostic tools for cancer. The Novel Diagnostics Program aims at exploiting the plentiful data stemming from “omics” projects to identify and develop new clinical markers for diagnosis and therapy response prediction in breast, prostate, bladder, and lung cancer patients. Recent results have been published by Dama et al. [131].

The overmentioned examples support the idea that the integrated work involving experts of different fields of the medical area will be decisive in the translation of the formulations described throughout this review into clinically used diagnostic tools.

## 4. Conclusions

Novel strategies to improve the imaging outcomes, leading to more precise and safe diagnostic procedures, are currently needed, and many Italian research groups offer a wide variety of examples in the field of imaging innovation.

Considering the works discussed in this review, it is evident that among the various applications, most attention is dedicated to the development of tools for cancer diagnosis.

Nanoparticulate systems seem to be among the most investigated because of their versatility and the possibility of being exploited for developing theranostic agents. Indeed, Italian research groups developed NPs as imaging agents detectable through different imaging techniques: in particular, nanosystems for PET detection (such as ^68^Ga-labeled carboxymethylcellulose NPs, ^18^F-labeled TSPO ligands, albumin and curcumin derivatives labeled with different radionuclides) are among the most studied, due to the high sensitivity characterizing this technique.

Moreover, combined imaging tools for multimodal imaging, such as the iron oxide NPs, SiNPs, and Fe-Au nanoalloys reported in the review, seem the most promising for future development. In fact, these multimodal systems, allowing to overcome and compensate for the limitations characterizing the clinically available detection techniques, might greatly increase the efficiency and quality of diagnostic outcomes. Indeed, the field of multimodal imaging represents an attractive area of research for many Italian groups.

## Figures and Tables

**Table 1:** Diagnostic systems developed by Italian research groups. The works reported in the table have been selected to showcase a range of recent advances from Italian research groups across the different areas of diagnostic medicine, favoring the most recent and/or the more advanced works.

Formulation Strategy	Formulation Innovation	Imaging Probe	Imaging Technique	Application	Tests	Ref.
Chemical modification, prodrugs and bio-precursors	Insertion of a deuterium atom in [^18^F]fluoromethyl-PBR28	^18^F	PET	Detection of inflammation associated to TSPO overexpression; diagnosis of neuroinflammation, neurodegeneration, and tumor progression	In vitro: human leukocyte membranes and rat cerebrocortical samples; Ex vivo: ICR mice In vivo: rat models of LPS-induced inflammation and of experimental autoimmune myocarditis	[24,25]
	Fluorine-substituted TSPO ligand	^18^F	PET	Detection of neuroinflammation, neurodegeneration, and tumor progression	Ex vivo: ICR mice In vivo: U87-MG xenografted Balb/c nu/nu mice	[17]
Use of buffers	Use of lactate buffer for [^18^F]F-DOPA dissolution	^18^F	PET	Detection of loss of functional dopaminergic neuron terminals in the striatum	In vitro: mouse skeletal muscle fibers In vivo: Wistar rats and C57BL/6J mice	[26]
Conjugates for active targeting	Synthesis of dextran-based multifunctional ligands	^99m^Tc	Lymphoscintigraphy	Sentinel lymph node detection for diagnosis of different types of cancers	In vitro: stability monitored in saline or serum	[27]
	Conjugation of an albumin derivative with a DOTA ring	^177^Lu, ^111^In, ^64^Cu	PET	Radio-guided occult lesion localization	In vivo: female adult rats	[28]
	Conjugation of a VCAM-1-binding peptide with a DOTA ring; development of a biotin/avidin three-step pretargeting system based on the same VCAM-1-binding peptide	^68^Ga	PET	Early diagnosis of atherosclerosis	In vitro: TNF-α activated HUVEC	[29,30]
	Conjugation of curcumin with a DOTA ring	^68^Ga	PET	Detection of colorectal cancer	In vitro: HT29 colorectal cancer cells In vivo: HT29 tumor-bearing mice	[31]
Nanoparticles	PAMAM dendrimer encapsulating sorafenib	FITC	Fluorescence optical imaging	Detection and treatment of liver cancer overexpressing ASGPR2	In vitro: HepG-2 human liver cancer cells	[32]
	PEG-decorated SLNs encapsulating Pt(IV)-prodrugs	Carbon dots	Fluorescence optical imaging	Assessment of BBB permeability	In vitro: hCMEC/D3 cells and polarized hCMEC/D3 endothelial cells seeded on a porous membrane (BBB model)	[33]
	PLGA-TSPO NPs	FITC	Fluorescence optical imaging	Subcellular targeting and imaging of TSPO-overexpressing cells	In vitro: rat C6 glioma cells	[34]
	TSPO-dextran nanogel	FITC	Fluorescence optical imaging	Subcellular targeting and imaging of TSPO-overexpressing cells	In vitro: rat C6 glioma cells	[35]
	PAMAM dendrimer	FITC	Fluorescence optical imaging	Detection of tumors or neurodegenerative diseases associated to TSPO overexpression	In vitro: C6 rat glioma cells	[36]
	Silica shell functionalized QDs	QDs	Fluorescence optical imaging	Detection of tumors or neurodegenerative diseases associated to TSPO overexpression	In vitro: C6 rat glioma cells	[37]
	USPIONs	Cyanine 5.5 fluorescent dye	NIR optical imaging	Detection of glioblastoma exploiting TSPO targeting	In vitro: U87-MG glioblastoma cells and PC3 prostate cancer cells In vivo: Balb/c athymic mice	[38]
	ECFC cells encapsulating AuNPs	AuNPs	PA imaging	Detection and treatment of cancer	In vitro: stack of chicken breast muscle Ex vivo: mouse melanoma, liver and spleen In vivo: CD1 immunodeficient mice	[39]
	Chitosan nanobubbles	Gd(III)-DOTP complex	MRI	Detection and treatment of cancer	In vitro: preliminary evaluation of US properties in agar gel suspension	[40]
	Fe-B NPs enveloped by polyvinylpyrrolidone	Iron	MRI	Optimization of NCT procedures for cancer treatment	In vitro: L929 fibroblasts, 4T1 mammary carcinoma cells and B16 melanoma cells In vivo: Balb/c mice	[41]
	Iron-doped hydroxyapatite NPs	Iron	MRI coupled to SPECT and/or PET	-	In vivo: C57BL/6 mice	[42]
	PLGA-PEG micelles encapsulating QDs	QDs	MRI	Detection of inflammatory bowel disease by MAdCAM-1 targeting	In vitro: SKBR3 cells In vivo: C57BL/6 mice	[43]
	Carboxymethylcellulose NPs	^68^Ga	PET	White blood cells imaging	In vitro: human leukocytes	[44]
	SiNPs	^99m^Tc	SPECT	Detection of HER2-positive breast cancer	In vitro: SK-BR-3 cells Ex vivo, in vivo: SK-BR-3 tumor-bearing mice	[45,46]
	Fe_3_O_4_ NPs	Fe_3_O_4_ and 7-nitrobenzofurazan fluorescent dye	Optical imaging/MRI	Detection and treatment of cancer	In vitro: preliminary evaluation of fluorescence and MRI properties	[47]
	SiNPs embedding dye-modified Au NPs	IRDye 800CW and AuNPs	US/PA imaging	Detection and treatment of cancer	Ex vivo: chicken breast samples	[48]
	Au-Fe nanoalloys	Au and Fe	X-ray CT/MRI	-	In vivo: Balb/c mice	[22]

## Data Availability

Not applicable.
